# Enantiomeric Composition, Antioxidant Capacity and Anticholinesterase Activity of Essential Oil from Leaves of Chirimoya (*Annona cherimola* Mill.)

**DOI:** 10.3390/plants11030367

**Published:** 2022-01-28

**Authors:** Eduardo Valarezo, Jeannette Ludeña, Estefanía Echeverria-Coronel, Luis Cartuche, Miguel Angel Meneses, James Calva, Vladimir Morocho

**Affiliations:** Departamento de Química, Universidad Técnica Particular de Loja, Loja 110150, Ecuador; isah4a@hotmail.com (J.L.); beecheverria@utpl.edu.ec (E.E.-C.); lecartuche@utpl.edu.ec (L.C.); mameneses@utpl.edu.ec (M.A.M.); jwcalva@utpl.edu.ec (J.C.); svmorocho@utpl.edu.ec (V.M.)

**Keywords:** essential oil, *Annona cherimola*, chemical composition, enantioselective analysis, antibacterial activity, antioxidant activity, anticholinesterase activity, germacrene D, *Campylobacter jejuni*

## Abstract

*Annona cherimola* Mill. is a native species of Ecuador cultivated worldwide for the flavor and properties of its fruit. In this study, hydrodistillation was used to isolate essential oil (EO) of fresh *Annona cherimola* leaves collected in Ecuadorian Sierra. The EO chemical composition was determined using a non-polar and a polar chromatographic column and enantiomeric distribution with an enantioselective column. The qualitative analysis was carried out by gas chromatography coupled to a mass spectrometer and quantitative analysis using gas chromatography equipped with a flame ionization detector. The antibacterial potency was assessed against seven Gram-negative bacteria and one Gram-positive bacterium. ABTS and DPPH assays were used to evaluate the radical scavenging properties of the EO. Spectrophotometric method was used to measure acetylcholinesterase inhibitory activity. GC-MS analysis allowed us to identify more than 99% of the EO chemical composition. Out of the fifty-three compounds identified, the main were germacrene D (28.77 ± 3.80%), sabinene (3, 9.05 ± 1.69%), β-pinene (4, 7.93 ± 0.685), (E)-caryophyllene (10.52 ± 1.64%) and bicyclogermacrene (11.12 ± 1.39%). Enantioselective analysis showed the existence of four pairs of enantiomers, the (−)-β-Pinene (1S, 5S) was found pure (100%). Chirimoya essential oil exhibited a strong antioxidant activity and a very strong anticholinesterase potential with an IC_50_ value of 41.51 ± 1.02 µg/mL. Additionally, EO presented a moderate activity against *Campylobacter jejuni* and *Klebsiella pneumoniae* with a MIC value of 500 μg/mL.

## 1. Introduction

Worldwide, the Annonaceae family comprises more than 128 genera and approximately 2106 species and they are mainly distributed in tropical and subtropical regions [1]. For Ecuador, 25 genera, 106 species and 20 endemic species are reported [2]. In a review of the antimalarial properties of the Annonaceae family, 11 species from *Annona* and *Xilopia* genus were recognized for their antiparasitic potential. Annonaceae species used in traditional medicine, over the tropical regions, are well documented for having potential for the treatment of parasitic diseases such as Malaria, Chagas and Leishmaniasis as well as other illnesses [3]. Indeed, *Annona muricata* was one of the most cited species with a variety of medicinal properties including the treatment for the symptoms of malarial infection, fever, liver ailments and headaches [4].

*Annona cherimola*, *A. crassiflora*, *A. muricata*, *A. squamosa* and *A. reticulata* are the commercial species, highly valued by their exotic edible fruit. Furthermore, different parts of the tree from these species have been used in folk medicine to treat several conditions including gastrointestinal diseases, diabetes and hypertension [5]. Many secondary metabolites have been reported such as phenols and other bioactive compounds but, the main chemical marker of the genus is a diverse group of polyketides called acetogenins, compounds closely associated to their antiproliferative effect on cancer cell lines [5,6].

In a related study, nine species from *Annona* genus, including *A. cherimola*, where reviewed in relation to their phytochemical composition and biological activity and found that mainly polar extracts obtained from these plants, induce a reduction in blood sugar levels in chemically induced type-2 diabetic rats which demonstrate the antidiabetic potential of species from this genus. Likewise, seven out of nine of the studied species reported a good antioxidant capacity profile in different in vitro assays. *A. cherimola* was only tested trough the oxygen radical absorbance capacity (ORAC) assay [7].

*Annona cherimola* Mill. is a native shrub, widely distributed in the Andean, Coastal, Amazon and Insular regions of Ecuador, between 0–3000 m a.s.l [2]. Currently, this species is cultivated in the subtropical and tropical regions worldwide, especially for its fruit, which is considered exotic. The *A. cherimola* species is commonly known as “chirimoya” or “chirimoyo” (Spanish language) and “custard-apple” (English language) [8]. The vernacular name “chirimoya” is derived from the Quechua (indigenous language) word “chirimuya”, “chiri” that means cold and “muya” seeds. The fruit of this species is considered one the most appreciated inside the genus. The plant has been used in traditional medicine for the treatment of boils and others skin diseases [9].

Anthropological evidence suggests that the species *A. cherimola* was cultivated since the times of the Incan Empire and its fruit was considered as an active ingredient in the their diet [10]. Some important compounds have been isolated from this species such as alkaloids as cherimoline, annocherine A, annocherine B, cherianoine and rumocosine H [11], and some amides as cherinonaine, cheritamide, (N-trans-feruloyltyramine, N-trans-caffeoyltyramine, N-*cis*-caeoyltyramine, dihydro feruloyltyramine, N-trans-feruloylmethoxytyramine, N-*cis*-feruloylmethoxytyramine, and N-*p*-coumaroyltyramine [12]. Recent studies have shown that custard apple leaves contain flavonoids and other phenolic compounds with biological properties [13,14] and that alcoholic extracts from the leaves have proapoptotic and antidepressant activities [15].

Several studies on the species of the *Annona* genus have reported the occurrence of compounds with potential application for pharmaceuticals, food, agrochemicals products and cosmetics [3,5,6,16]. Extracts from these species are used for a wide range of beneficial purposes, however, the research have been focused mainly on the non-volatile fraction of the fruits, meanwhile, the enantiomeric distribution and biological properties of the EOof *Annona cherimola* have not been reported previously. This fact stimulated our interest in studying the chemical composition, enantiomeric distribution and antimicrobial, antioxidant and anticholinesterase activities of the essential oil of custard apple leaves.

## 2. Results

A total of 15,150 g of fresh custard apple leaves, with a moisture of 53 ± 5% (*w/w*), were used as raw material for the extraction. The isolation of the essential oil was carried out by hydrodistillation using a Clevenger-type apparatus. The amount of EO obtained was 38.5 mL, which represents a yield of 0.25 ± 0.02 (*v/w*).

### 2.1. Physical Properties of Essential Oil

The essential oil isolated from leaves of *A. cherimola* was presented as a viscous liquid with a characteristic texture. The physical properties of EO are shown in Table 1. In addition, this table shows the subjective color and its RGB and CMYK values.

### 2.2. Chemical Constituents of the Essential Oil

The compounds ocurring in *A. cherimola* EO were identified and quantified by GC-MS and GC-FID using nonpolar and polar columns. The Table 2 shows the quantitative and qualitative data of chemical constituents of custard apple obtained using nonpolar column DB-5ms. In the essential oil from chirimoya leaves fifty-three compounds were identified, which represent 99.80% of the total composition. According to their chemical nature, all the compounds were grouped in aliphatic monoterpene hydrocarbons (ALM), oxygenated monoterpenes (OXM), aliphatic sesquiterpene hydrocarbons (ALS) and oxygenated sesquiterpene (OXS). The ALS were the most representative compounds with twenty-seven compounds, which represents 69.40%, followed by ALM with 25.68%. Compounds belonging to the aromatic monoterpene hydrocarbons, aromatic sesquiterpene hydrocarbons and oxygenated sesquiterpene groups were not identified. The ALS germacrene D (compound 32, CF: C_15_H_24_, MM: 204.19 Da) was the main constituent with 28.77 ± 3.80%. Other main compounds (>5%) were sabinene (3, 9.05 ± 1.69%), β-pinene (4, 7.93 ± 0.68%), (E)-caryophyllene (24, 10.52 ± 1.645) and bicyclogermacrene (36, 11.12 ± 1.39%). Compounds 8 and 9 (limonene and β-phellandrene) co-eluted, both representing 0.66 ± 0.13%.

Coeluting compounds (limonene and β-phellandrene) were separated using an HP-INNOWax polar column. The retention index in this column for limonene was 1192 and for β-phellandrene was 1201. Limonene (mixture of (+)-limonene and (−)-limonene) presented a percentage of 0.55 ± 0.09% and β-phellandrene a value of 0.12 ± 0.01%.

### 2.3. Enantioselective Analysis

The enantioselective analysis from *Annona cherimola* EO was achieved for the first time. The Table 3 shows the enantiomeric distribution, linear retention indices and enantiomeric excess (e.e.) of each enantiomer. Using a chiral column could be quantified four pairs of enantiomers, whose peaks were well separated at the base. The β-pinene (−) was found practically pure, while (−)-α-pinene and (−)-sabinene and (−)-germacrene D exhibited a high enantiomeric excess, whereas (−)-limonene and (+)-limonene were found in a racemic mixture.

### 2.4. Antibacterial Activity

Microdilution broth method was used to assess the antibacterial activity of essential oil of *A. cherimola* leaves. Tetracycline was used as a positive control and the maximum evaluated concentration was 1000 µg/mL. The minimum inhibitory concentration (MIC) values and the microorganisms used (seven Gram-negative bacteria and one Gram-positive bacterium) are shown in Table 4. The *A. cherimola* essential oil reported MIC values of 500 µg/mL against *Campylobacter jejuni* (ATCC 33560). EO dissolved in aqueous media caused the formation of an emulsion that difficulted the visual observation of bacterial growth particularly with *C. jejuni.* The reduction of 2,3,5-Triphenyl tetrazolium chloride (TTZ) yield a red color product only in the wells were bacteria developed a well growth. A blank of EO with the same range of concentrations and media was included to discard interferences due to contamination which was also confirmed by reading at 405 nm (data not shown) For the other bacteria, the essential oil did not show activity at the maximum dose tested.

### 2.5. Antioxidant Capacity

The results obtained for DPPH and ABTS radical scavenging of the EO are shown in Table 5. The results are expressed as the concentration of the EO that scavenge or decrease the concentration of the radical at 50% (SC_50_). Trolox was used as a positive control.

**Figure 1:**
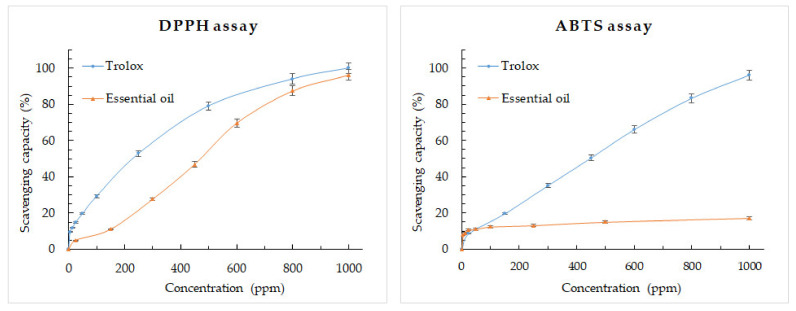
Scavenging capacity vs. concentration of *Annona cherimola* essential oil obtained by DPPH and ABTS assays.

Through the DPPH method, the essential oils of *A. cherimola* showed strong antioxidant activity with a SC_50_ value of 470 ± 30 μg/mL. Employing the ABTS technique the SC_50_ could not be calculated at the concentration ranges tested (Figure 1).

### 2.6. Anticholinesterase Activity

**Figure 2:**
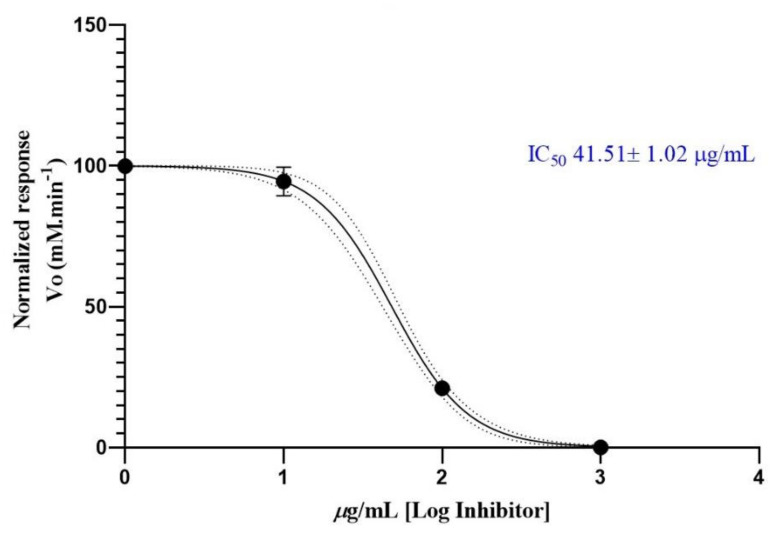
Half-maximum inhibitory concentration of *Annona cherimola* essential oil against acethylcholinesterase.

Three different concentrations of the essential oil from *A. cherimola* leaves were used to determine its anticholinesterase potential. The data obtained by measuring the rate of reaction of AChE against EO are shown in Figure 2. The results plotted as Log (concentration essential oil) vs. normalized response rate of reaction allowed us to calculate the IC_50_ value. The IC_50_ value obtained for chirimoya essential oil was 41.51 ± 1.02 µg/mL. The positive control (donepezil) exhibited an IC_50_ value of 13.80 ± 1.01 nM.

## 3. Discussion

The essential oil from *Annona cherimola* exhibited a low yield of 2.5 ± 0.2 mL/Kg [17]. The extraction yield of essential oils is very variable between plant species and depends on different aspects related to the plant such as the part, the age and the time after plant collection and other aspects related to the isolation process such as the pretreatment of the material (drying, grinding, etc.) and the extraction time [18].

The aroma of the *Annona* species is well recognized and has been studied in some species, however, little has been reported about the essential oil composition of *Annona cherimola*. In the present study, the main chemical components identified were aliphatic monoterpenes (25.68%) and aliphatic sesquiterpenes (69.40%), which was similar to the information reported by Rabelo et al. [19]. Furthermore, Rios et al. in 2003 [20] reported monoterpenes (6.09%) and sesquiterpenes (76.56%) as the main type of compounds in the *A. cherimola* EO. On the other hand, the same type of volatile compounds were meaningful in fruits of *Annona cherimola* (monoterpene 40.3% and sesquiterpene 24.3%) [16].

The major components (>5%) identified in the *A. cherimola* EO were germacrene D (28.77%), bicyclogermacrene (11.12%), (E)-caryophyllene (10.52%), sabinene (9.05%) and β-pinene (7.93%). The results are different to those reported by Elhawary et al. fβ-caryophyllene with 9.50%, germacrene-D with 17.71% an β-elemene with 25.02% [21], and those reported by Rios et al. reported bicyclogermacrene (18.20%), trans-caryophyllene (11.50%), α-amorphene (7.57%), α-copaene (5.63%) and germacrene D (3.75%) [20]. In addition, Pino observed that the major compounds were α-thujene (18.7 ppm), α-pinene (23 ppm), terpinen-4-ol (19.8 ppm) and germacrene D (17.6 ppm) [16]. Despite the differences in their concentrations, the main component that is common in all the studies is germacrene D. It is well known the influence of different cultivation and climatic factors over the chemical composition of the essential oils.

Due to the relevance of aromatic compounds of the *Annona* species Ferreira et al. in 2009 compared the essential oil and the volatile compounds of the leaves and fruits of *Annona cherimola*. The chemical composition for the EO was different to the volatile compounds in fruits, the main compounds in the leaves essential oil were identified in lower quantities, germacrene-D (0.11% to 0.22%), sabinene (not identified), β-pinene (0.79% to 3.60%), (E)-caryophyllene (0.23% to 0.32%) and bicyclogermacrene (not identified) while the main compounds analyzed by headspace solid phase microextraction were methyl butanoate, butyl butanoate, 3-methylbutyl butanoate, 3-methylbutyl 3-methylbutanoate and 5-hydroxymethyl-2-furfural [22].

This is the first report of enantioselective GC-MS analysis of *A. cherimola* EO, this analysis showed the ocurrence of five pairs of enantiomers and one enantiomerically pure chiral monoterpenoid, β-pinene. The enantiomeric ratio of an essential oil is an important information which could be related with the biological activity, metabolism and organoleptic quality of the enantiomeric pairs [23]. The enantiomeric excess (e.e %) were (−)-α-pinene (1S,5S) (63.99%), (−)-sabinene (1S,5S) (95.91%), (−)-limonene (4R) (27.50%) and (−)-germacrene D (8S) (95.91%).

Regarding their biological activity, the essential oil of *Annona cherimola* showed moderate antibacterial activity against *Campylobacter jejuni* (ATCC 33560) and *Klebsiella pneumonia* (ATCC 9997), both with MIC at 500 μg/mL and no activity for the other bacteria tested (MIC was higher than 1000 μg/mL). Compared to data reported in the literature, Rios et al. in 2003 reported a significant activity against two Gram-positive bacteria *Staphylococcus aureus* (MIC 250 μg/mL) and *Enterococcus faecalis* (MIC 500 μg/mL), however, the MIC values for Gram-negative bacteria were higher than 5000 μg/mL [20]. Elhawary et al. in 2013 reported the MIC of EO *A. cherimola* for *Bacillus subtilis* (130 μg/mL), *Staphylococcus aureus* (285 μg/mL), *Escherichia coli* (110 μg/mL), *Pseudomonas aeruginosa* (140 μg/mL), and *Candida albicans* (152 μg/mL) [21]. When the antibacterial activity of pure compounds was analyzed [20] the MIC of trans-caryophyllene, β-pinene, linalool, and other compounds was higher than the value for the essential oil, therefore suggesting that the antibacterial potency could be exerted by a synergistic effect among the constituents above mentioned. The essential oil of *Annona* species showed a wide range of biological activity, for *A. vepretorum* Costa et al. in 2012 reported a moderate activity (MIC 500 μg/mL) against *Staphylococcus aureus* and *Staphylococcus epidermis* and a significant activity against *Candida tropicalis* (MIC 100 μg/mL) [24]. Another study, in 2013, Costa et al. observed the antibacterial activity of essential oil of *A. salzmannii* and *A. pickelii* against *Staphylococcus aureus*, *Staphylococcus epidermis* and *Candida tropicalis* with MIC of 500 μg/mL [25].

Some studies have shown that the enantiomers of a compound have different biological activities. Lis-Balcnin et al. reported that 18 out of 25 different bacteria were more affected by the (−)-α-pinene in comparison with the (+) enantiomer, 19 out of 20 different *Listeria monocytogenes* strains were affected more by (+)-α-pinene isomer and two of three filamentous fungi were affected more by the (+) enantiomer [26]. The MIC and minimal microbicidal concentration (MMC) showed that the positive enantiomers of pinene exerted a microbicidal effect against all the fungi and bacteria tested with MIC values ranging from 117 to 4150 µg/mL. However, with concentrations up to 20 mg/mL of the negative enantiomers, no antimicrobial activity was observed [27]. The MIC values against three Gram-positive (*B. cereus*, *E. faecalis* and *S. aureus*) and four Gram-negative (*E. coli*, *K. pneumoniae*, *M. catarrhalis* and *P. aeruginosa*) bacteria were in the ranges of 3 to 27 mg/mL for (+)-limonene and 2 to 27 mg/mL for (−)-limonene. The greatest difference was obtained against *Staphylococcus aureus* ATCC 12600 where the (+)-limonene showed a MIC of 14 mg/mL and the (−)-limonene a MIC of 4 mg/mL [28]. Omran et al. found that (−)-limonene had better antifungal activity than (+)-limonene against *Aspergillus niger*, *Aspergillus* sp., *Candida albicans* and *Penicillium* sp. [29]. It was not possible to find previous studies about the antifungal or antibacterial activity of the (+) and (−) enantiomers of the main compound germacrene D, however, Stranden et al. determined that the two enantiomers of this compound mediate the same kind of information to the receptor neurons of the moth *Helicoverpa armigera*, but (−)-germacrene D had approximately 10 times stronger effect than (+)-germacrene D [30]. The difference in biological activity of the enantiomers is maintained even when they are mixed with other compounds [28]. The enantiomers of a compound have different biological activities, then, the enantiomeric distribution of the compounds could influence the biological activity for an essential oil.

Regarding their antioxidant effect, the *Annona cherimola* essential oil showed an SC_50_ of 470 μg/mL for the DPPH assay while the SC_50_ was >1000 μg/mL in the ABTS assay. Costa et al. reported as strong the antioxidant activity of EO *A. salzamannii* and *A. pickelii* measured by a TLC-based DPPH assay, however, the individual components β-pinene and α-pinene did not show antioxidant activity [25]. Araújo, et al. [31] and Costa, et al. [24] reported a weak antioxidant activity for the EO of *A. vepretorum*. Another study, Gyesi, et al. in 2019 [32] reported an SC_50_ of 244.8 μg/mL from the DPPH assay for the EO of *A. muricata*. The differences between the antioxidant activity of EO and pure compounds could correspond to synergistic effects among the components in the essential oil.

The acetylcholinesterase inhibitory activity of *A. cherimola* EO has not been previously reported. Chirimoya EO showed an AChE IC_50_ value of 41.51 μg/mL, this inhibitory activity could be considered very strong compared to the related EO of *Piper carpunya* (IC_50_ of 36.42 μg/mL) [33]. The inhibition of AChE due to EO is of relevant interest in the treatment of Alzheimer disease since different studies report in vitro and clinical AChE inhibitory activity. Benny and Tomas summarize the neuroprotective effects of EO and its relevance on Alzheimer disease stating that EO could rebuild the antioxidant status of brain which confer neuroprotective effect as in the case of EO of *Coriandrum sativum* L., *Syzygium aromaticum* (L.), *Juniperus communis*, *Rosmarinis officinalis* (L.), and other species. The same activity has been observed for pure compounds such as thymol, linalool, α-terpinene, α-terpineol, carvacrol, (E)-β-caryophyllene, α-pinene, and eugenol [34].

## 4. Materials and Methods

### 4.1. Materials

Dichloromethane (DMC), methanol (MeOH), sodium sulfate anhydrous, DPPH (2,2-diphenyl-1-picrylhydryl), ABTS (2,2′-azinobis-3-ethylbenzothiazoline-6-sulfonic acid), acetylcholinesterase (AChE), acetylthiocholine (ATC), phosphate buffered saline, Ellman’s reagent (donepezil, 5,5′-dithiobis(2-nitrobenzoic acid)), tris hydrochloride (Tris-HCl), magnesium chloride hexahydrate and 2,3,5-Triphenyl tetrazolium chloride (TTZ) were purchased from Sigma-Aldrich (San Luis, MO, USA). The microbiological media such as Mueller Himton broth, Mueller Hinton II broth and fluid thioglycollate medium were purchased from DIPCO (Quito, Ecuador). Horse serum and Oxoid CampyGen were purchased from Thermo Fisher Scientific (Waltham, MA, USA). The standard aliphatic hydrocarbons were purchased from ChemService (West Chester, PA, USA). Helium was purchased from INDURA (Quito, Ecuador). All chemicals were of analytical grade and used without further purifications.

### 4.2. Plant Material

*Annona cherimola* leaves were collected under permission granted for Ministerio del Ambiente de Ecuador (Ecuadorian Environmental Ministry) by means of the authorization No. 001-IC-FLO-DBAP-VS-DRLZCH-MA. The leaves of chirimoya were collected between the months of October and January in the surroundings of the locality of Cango Bajo, canton Calvas, province of Loja, Ecuador, at 1950 m a.s.l. at a latitude of 4°20′34″ S and a longitude of 79°34′24″ W. Collection, store and transfer of chirimoya leaves were performed according to what is described by Valarezo et al. [35]. Botanist Nixon Cumbicus made the identification of the plant material. A voucher specimen was deposited at the Herbarium of Universidad Técnica Particular de Loja (HUTPL).

### 4.3. Postharvest Treatments

Between 2 and 3 h after being collected, the plant material was subjected to the postharvest treatments, which consist of the separation of degraded leaves and foreign material.

### 4.4. Moisture Determination

Method Loss on drying (Moisture) in plants, AOAC 930.04-1930, was used to determine the moisture of plant material, for this an analytical balance (Mettler AC 100, Mettler Toledo, Columbus, OH, USA) was used. Moisture was calculated according to Equation (1).

(1) $$\mathrm{Moisture}\;\left(\%\right)=\frac{\mathrm{wi}-\mathrm{wo}}{\mathrm{wi}}*100$$

where wi is the initial weight of sample and wo is weight of sample after drying.

### 4.5. Essential Oil Extraction

The isolation of the essential oil from leaves of *A. cherimola* was carried out by hydrodistillation using a Clevenger-type apparatus according to the procedure described by Valarezo et al. [36]. After being collected, the essential oil was dried using anhydrous sodium sulphate. Finally, the EO was stored at 4 °C in amber sealed vials until being used in the subsequent analysis.

### 4.6. Determination of the Physical Properties of the Essential Oil

Density of the essential oil was determined using the ISO 279:1998 standard (equivalent to the AFNOR NF T 75-111 standard). Density measurement was performed using an analytical balance (Mettler AC 100, Mettler Toledo, Columbus, OH, USA) and a pycnometer of 1 mL. Refractive index was determined using the standard ISO 280:1998 [37] (similary to AFNOR NF T 75-112), for which a refractometer (model ABBE, BOECO, Hamburg, Germany) was used. An automatic polarimeter (Mrc-P810, MRC, Holon, Israel) was used to measure the optical rotation of the EO according to the standard ISO 592:1998. All measurements were taken at 20 °C.

### 4.7. Identification of the Chemical Constituents of the Essential Oil

#### 4.7.1. Quantitative and Qualitative Analysis

The dentification of the chemical constituents of the essential oil was carried out using an Agilent gas chromatograph (GC) (6890N series, Agilent Technologies, Santa Clara, CA, USA). For the quantitative analysis gas chromatograph was equipped with a flame ionization detector (FID) and for qualitative analysis gas chromatograph was coupled to a mass spectrometer (quadrupole) detector (MS) (model Agilent 5973 inert series, Agilent Technologies, Santa Clara, CA, USA). The GC-FID and GC-MS analyses were performed according to the procedure described by Valarezo et al. [35]. The injection of the samples was carried out by an automatic injector (Agilent 7683 automatic liquid sampler, Agilent Technologies, Santa Clara, CA, USA) in split mode. Chromatographic runs were performed using a nonpolar and a polar column. The nonpolar was an Agilent J&W DB-5ms Ultra Inert GC column with stationary phase 5%-phenyl-methylpolyxilosane and the polar was an Agilent J&W HP-INNOWax GC column with stationary phase polyeth-ylene glycol. Both columns with a length of 30 m, an outer diameter of 0.25 mm and a stationary phase thickness of 0.25 µm. Identification of the EO compounds was based on a comparison of relative retention indices (RIs) and mass spectra data with those of the published literature [38,39] according as described by Valarezo et al. [36].

#### 4.7.2. Enantioselective Analysis

The enantiomeric distribution was performed using an enantioselective column with stationary phase 2,3-diethyl-6-tert-butyldimethylsilyl-β-cyclodextrin. The chromatographic run was performed with a temperature ramp of 2 °C/min from 50 °C (maintained for 2 min) to 220 °C (maintained for 2 min) in an Agilent gas chromatograph (model 6890N series, Agilent Technologies, Santa Clara, CA, USA) coupled to a mass spectrometer (quadrupole) detector (model Agilent 5973 inert series, Agilent Technologies, Santa Clara, CA, USA). The injection of enantiomerically pure standards was used to determine the order of elution of the enantiomers.

### 4.8. Evaluation of Antibacterial Activity

The antibacterial activity of the essential oil from chirimoya leaves was assessed against Gram-negative and Gram-positive bacteria (Table 4) by the microdilution broth method according to the procedure described by Valarezo et al. [40]. The bacterial strains were incubated in Müeller-Hinton (MH) broth. Tetracycline was used as a positive control and DMSO was used as a negative control. Results are reported as minimum inhibitory concentration (MIC).

For *Campylobacter jejuni* (ATCC 33560) the broth microdilution method was carried out according to Valarezo et al. [40] with some specific requirements as described briefly. Fluid thioglycollate medium was used for reactivation of the strain, supplemented with 5% of Horse serum. An aliquot of a cryogenic reserve was resuspended in thioglycollate and incubated for 48 h at 37 °C in a microaerophilic atmosphere provided by an Oxoid CampyGen (2.5 L sachet). Sample solutions were made by dissolving 80 mg of EO in 1 mL of DMSO. Two-fold serial dilutions were employed to obtain decreasing concentrations of EO from 4000 to 31.25 µg/mL and cation-adjusted Muller Hinton II broth (pH 7.3) with 5% lyssed horse blood [41] as media for the antibacterial assay. The inoculum was prepared from thioglycollate culture and adjusted to 0.5 McFarland. Final concentration of bacteria was 5 × 10^5^ CFU/mL. The microplate was incubated for 48 h at 37 °C in microaerophilic atmosphere (5% CO_2_, Oxoid CampyGen). Erythromycin was used as positive control with a MIC value of 15.65 µg/mL and DMSO as negative control. MIC was determined by visual examination of growth and through addition of a 1% solution of TTZ as bacterial viability indicator after incubation time to confirm the visual results. A blank with the same range of concentrations of EO was prepared simultaneously and measurements at 405 nm were made to discard reduction of TTZ by contamination. Optical density (OD) for TTZ reduction was measured in a microplate reader (EPOCH 2, BioTek, Winooski, VT, USA).

### 4.9. Antioxidant Capacity

#### 4.9.1. DPPH Radical Scavenging Capacity

The DPPH assay was performed using 2,2-diphenyl-1-picrylhydryl free radical (DPPH^•^) based on the technique described by Brand Williams et al. [42] and Thaipong et al. [43] according to what was described by Valarezo et al. [33]. The concentrations of the EO from *A. cherimola leaves* used were 1000, 800, 600, 450, 300, 150 and 25 ppm. Trolox was used as a positive control and methanol as a blank control. The samples were evaluated in a UV spectrophotometer (Genesys 10S UV.Vis Spectrophotometer, Thermo Scientific, Waltham, MA, USA) at a wavelength of 515 nm. The percentage of scavenging capacity was calculated according to Equation (2). SC_50_ is the EO concentration that provided 50% DPPH^•^ scavenging effect.

(2) $$\mathrm{SC}\left(\%\right)=\frac{\left(\mathrm{AEO}-\;\mathrm{AMeOH}\right)}{\mathrm{AEO}}*100$$

where AEO is the absorbance of DPPH^•^ mixed with EO and As is absorbance of DPPH mixed with methanol.

#### 4.9.2. ABTS Radical Cation Scavenging Capacity

The ABTS assay was performed using 2,2′-azinobis-3-ethylbenzothiazoline-6-sulfonic acid radical cation (ABTS^•+^) according to the procedure report by Arnao et al. [44] and Thaipong et al. [43], as described by Valarezo et al. [33]. The concentrations of the essential oil from *A. cherimola* used were 1000, 500, 250, 100, 50 and 25 ppm. The samples were evaluated in a UV spectrophotometer (Genesys 10S UV.Vis Spectrophotometer, Thermo Scientific, Waltham, MA, USA) at a wavelength of 734 nm. Deionized water was used as a blank control and trolox was used as a positive control. The percentage of scavenging capacity was calculated according to equation.

(3) $$\mathrm{Sc}\left(\%\right)=\frac{\left(\mathrm{ASO}-\mathrm{ASA}\right)}{\mathrm{ASA}}*100$$

where ASO is the absorbance of ABTS^•+^ with solvent mixture and Ai is the absorbance after reaction of ABTS^•+^ with the sample.

### 4.10. Anticholinesterase Activity

The AChE inhibitory effect was measured based on the methodology designed by Ellman, et al. [45], with slight modifications as suggested by Rhee, et al. [46], as previously described by Valarezo et al. [33]. The inhibition of AChE was detected after the addition of Acetylthiocholine as the enzyme substrate and several concentrations of EO dissolved in MeOH. The enzyme reaction was monitored in a microplate reader (EPOCH 2, BioTek, Winooski, VT, USA) at 405 nm for 60 min. Final concentrations of 1000, 100, and 10 µg/mL of the EO in MeOH were prepared to assess the enzyme inhibition. The assay was carried out by triplicate in 96-well microplates. Donepezil was used as positive control. The IC_50_ value was calculated from the progression curve with the Graph Pad Prism software (v8.0.1.5., Graph Pad, San Diego, CA, USA)

### 4.11. Statistical Analysis

All procedures were repeated three times, except for the biological activity, which was repeated nine times. Microsoft Excel was used to collect the data and Minitab 17 (Version 17.1.0., Minitab LLC., State College, PA, USA) was used to calculate the measures of central tendency. The results are expressed as mean values.

## 5. Conclusions

Enantiomeric distribution, antibacterial activity against *Campylobacter jejuni*, antioxidant and anticholinesterase activities of the essential oil from *Annona cherimola* leaves were determined for the first time, in addition, the chemical composition and physical properties of this essential oil was studied. This research contributes to our knowledge about native species of Ecuador. The results obtained in this study motivate us to carry out new investigations in endemic and native species of this megadiverse country.

## Figures and Tables

**Table 1:** Physical properties of the essential oil.

	*Annona cherimola* EO	
	Mean ^a^	SD ^b^
Density, ρ (g/cm^3^)	0.9472	0.0044
Refractive index, *n*^20^	1.4713	0.0023
Specific rotation, [α] (°)	−61.8	0.8
Subjective color	Light-yellow	
RGB color values	R:255, G:255, B:224	
CMYK color values	C:0, M:0, Y:0.12, K:0	

^a^ Mean of nine determinations: three distillations × three collections, ^b^ Standard deviation.

**Table 2:** Chemical composition of essential oil from *Annona cherimola* leaves.

CN	RT	Compounds	RI	RI ^ref^	*A. chirimola* EO		Type	CF	MM (Da)
					%	SD			
1	5.73	α-Thujene	924	924	0.89	0.15	ALM	C_10_H_16_	136.1
2	5.94	α-Pinene	932	932	4.02	0.36	ALM	C_10_H_16_	136.1
3	7.37	Sabinene	969	969	9.05	1.69	ALM	C_10_H_16_	136.1
4	7.50	β-Pinene	973	974	7.93	0.68	ALM	C_10_H_16_	136.1
5	8.13	Myrcene	987	988	0.78	0.19	ALM	C_10_H_16_	136.1
6	8.72	α-Phellandrene	1001	1002	0.11	0.03	ALM	C_10_H_16_	136.1
7	9.15	α-Terpinene	1013	1014	0.14	0.04	ALM	C_10_H_16_	136.1
8, 9	9.64	Limonene + β-Phellandrene^a^	1023	1024	0.66	0.13	ALM	C_10_H_16_	136.1
10	10.08	(Z)-β-Ocimene	1032	1032	0.84	0.23	ALM	C_10_H_16_	136.1
11	10.54	(E)-β-Ocimene	1043	1044	0.73	0.12	ALM	C_10_H_16_	136.1
12	10.93	γ-Terpinene	1053	1054	0.46	0.13	ALM	C_10_H_16_	136.1
13	12.15	Terpinolene	1083	1086	0.07	0.01	ALM	C_10_H_16_	136.1
14	13.10	Linalool	1099	1095	0.17	0.01	OXM	C_10_H_18_O	154.1
15	16.55	Terpinen-4-ol	1176	1174	0.07	0.01	OXM	C_10_H_18_O	154.1
16	23.00	Bicycloelemene	1332	1330	0.33	0.07	ALS	C_15_H_24_	204.2
17	23.17	δ-Elemene	1337	1335	1.26	0.41	ALS	C_15_H_24_	204.2
18	23.66	α-Cubebene	1348	1345	0.46	0.11	ALS	C_15_H_24_	204.2
19	24.81	α-Copaene	1377	1374	3.06	0.77	ALS	C_15_H_24_	204.2
20	25.17	β-Panasinsene	1384	1381	0.14	0.04	ALS	C_15_H_24_	204.2
21	25.38	β-Cubebene	1389	1387	1.38	0.27	ALS	C_15_H_24_	204.2
22	25.49	β-Elemene	1391	1389	2.41	0.62	ALS	C_15_H_24_	204.2
23	25.99	(Z)-Caryophyllene	1406	1408	0.04	0.01	ALS	C_15_H_24_	204.2
24	26.59	(E)-Caryophyllene	1419	1417	10.52	1.64	ALS	C_15_H_24_	204.2
25	27.05	β-Gurjunene	1429	1431	0.10	0.02	ALS	C_15_H_24_	204.2
26	27.16	γ-Elemene	1432	1434	0.20	0.04	ALS	C_15_H_24_	204.2
27	27.59	α-Guaiene	1439	1437	0.12	0.03	ALS	C_15_H_24_	204.2
28	27.86	Aromadendrene	1443	1439	0.33	0.08	ALS	C_15_H_24_	204.2
29	28.06	α-Humulene	1453	1452	2.05	0.51	ALS	C_15_H_24_	204.2
30	28.22	allo-Aromadendrene	1457	1458	0.20	0.03	ALS	C_15_H_24_	204.2
31	28.78	4,5-di-epi-Aristolochene	1470	1471	tr	-	ALS	C_15_H_24_	204.2
32	29.01	γ-Gurjunene	1475	1475	0.45	0.07	ALS	C_15_H_24_	204.2
33	29.20	Germacrene D	1478	1480	28.77	3.80	ALS	C_15_H_24_	204.2
34	29.46	β-Selinene	1486	1489	0.38	0.12	ALS	C_15_H_24_	204.2
35	29.56	γ-Amorphene	1493	1495	0.20	0.08	ALS	C_15_H_24_	204.2
36	29.74	Bicyclogermacrene	1497	1500	11.12	1.39	ALS	C_15_H_24_	204.2
37	29.96	α-Muurolene	1501	1500	0.39	0.07	ALS	C_15_H_24_	204.2
38	30.17	Germacrene A	1506	1508	1.37	0.35	ALS	C_15_H_24_	204.2
39	30.49	δ-Amorphene	1510	1511	0.41	0.07	ALS	C_15_H_24_	204.2
40	30.63	γ-Cadinene	1512	1513	0.37	0.11	ALS	C_15_H_24_	204.2
41	30.73	δ-Cadinene	1517	1522	1.79	0.39	ALS	C_15_H_24_	204.2
42	31.26	trans-Cadina-1,4-diene	1530	1533	0.09	0.02	ALS	C_15_H_24_	204.2
43	32.03	Elemol	1546	1548	0.13	0.07	OXS	C_15_H_26_O	222.2
44	32.14	Germacrene B	1554	1559	1.45	0.24	ALS	C_15_H_24_	204.2
45	32.75	(E)-Nerolidol	1563	1561	0.54	0.08	OXS	C_15_H_26_O	222.2
46	32.97	Germacrene D-4-ol	1571	1574	1.41	0.42	OXS	C_15_H_26_O	222.2
47	33.45	Carotol	1590	1594	0.08	0.02	OXS	C_15_H_26_O	222.2
48	33.70	Ledol	1597	1602	1.12	0.17	OXS	C_15_H_26_O	222.2
49	34.75	1-epi-Cubenol	1621	1627	0.18	0.02	OXS	C_15_H_26_O	222.2
50	35.52	cis-Cadin-4-en-7-ol	1629	1635	0.12	0.01	OXS	C_15_H_26_O	222.2
51	35.59	epi-α-Cadinol	1632	1638	0.33	0.08	OXS	C_15_H_26_O	222.2
52	36.00	α-Muurolol (=Torreyol)	1640	1644	0.56	0.14	OXS	C_15_H_26_O	222.2
53	36.61	α-Cadinol	1653	1652	tr	-	OXS	C_15_H_26_O	222.2
ALM					25.68				
OXM					0.24				
ALS					69.40				
OXS					4.47				
Total identified					99.80				

RT: Retention Time; RI: Calculated Retention Indices; RI ^ref^: References Retention Indices; SD: Standard Deviation; CF: Chemical Formula; MM: Monoisotopic Mass; tr: traces; -: not calculated. ^1^ Co-eluted compounds.

**Table 3:** Enantiomeric distribution of chiral constituents occurring in the EO of *A. cherimola*.

Enantiomers	RT	RI	Enantiomeric Distribution	e.e.
	min		%	%
(+)-α-Pinene (1R,5R)	4.60	916	18.01	63.99
(−)-α-Pinene (1S,5S)	5.10	921	81.99	
(−)-β-Pinene (1S,5S)	10.01	970	100.00	100.00
(+)-Sabinene (1R,5R)	10.51	975	2.05	95.91
(−)-Sabinene (1S,5S)	10.91	979	97.95	
(−)-Limonene (4S)	16.72	1037	63.75	27.50
(+)-Limonene (4R)	17.22	1042	36.25	
(+)-Germacrene D (8R)	61.61	1485	2.05	95.91
(−)-Germacrene D (8S)	62.01	1489	97.95	

**Table 4:** Antibacterial activity of essential oil from *Annona cherimola* leaves.

Bacteria	*Annona cherimola*	Positive Control ^a^
	MIC (μg/mL)	
Gram-negative		
*Campylobacter jejuni* (ATCC 33560)	500	15.65
*Escherichia coli* (ATCC 25922)	>1000	1.95
*Klebsiella pneumoniae* (ATCC 9997)	500	1.95
*Proteus vulgaris* (ATCC 8427)	>1000	7.81
*Pseudomonas aeruginosa* (ATCC 27853)	>1000	15.62
*Salmonella typhimurium* (LT2)	>1000	3.90
*Salmonella enterica* (ATCC 29212)	>1000	1.95
Gram-positive		
*Staphylococcus aureus* (ATCC 25923)	>1000	1.95

^a^ Erythromycin for *Campylobacter jejuni* and tetracycline for other bacteria.

**Table 5:** Antioxidant activity of essential oils of *Annona cherimola*.

Sample	DPPH	ABTS
	SC_50_ (μg/mL)	
*Essential oil*	470 ± 30	>1000
Trolox	232 ± 20	446 ± 30

## Data Availability

Data are available from the authors upon reasonable request.
